# Supplementary figures and images for: The nuclear localization signal of CPSF6 governs post-nuclear import steps of HIV-1 infection
Source: PLoS Pathog. 2025 Jan 17;21(1):e1012354. doi: 10.1371/journal.ppat.1012354 (PMC11844840; doi:10.1371/journal.ppat.1012354)

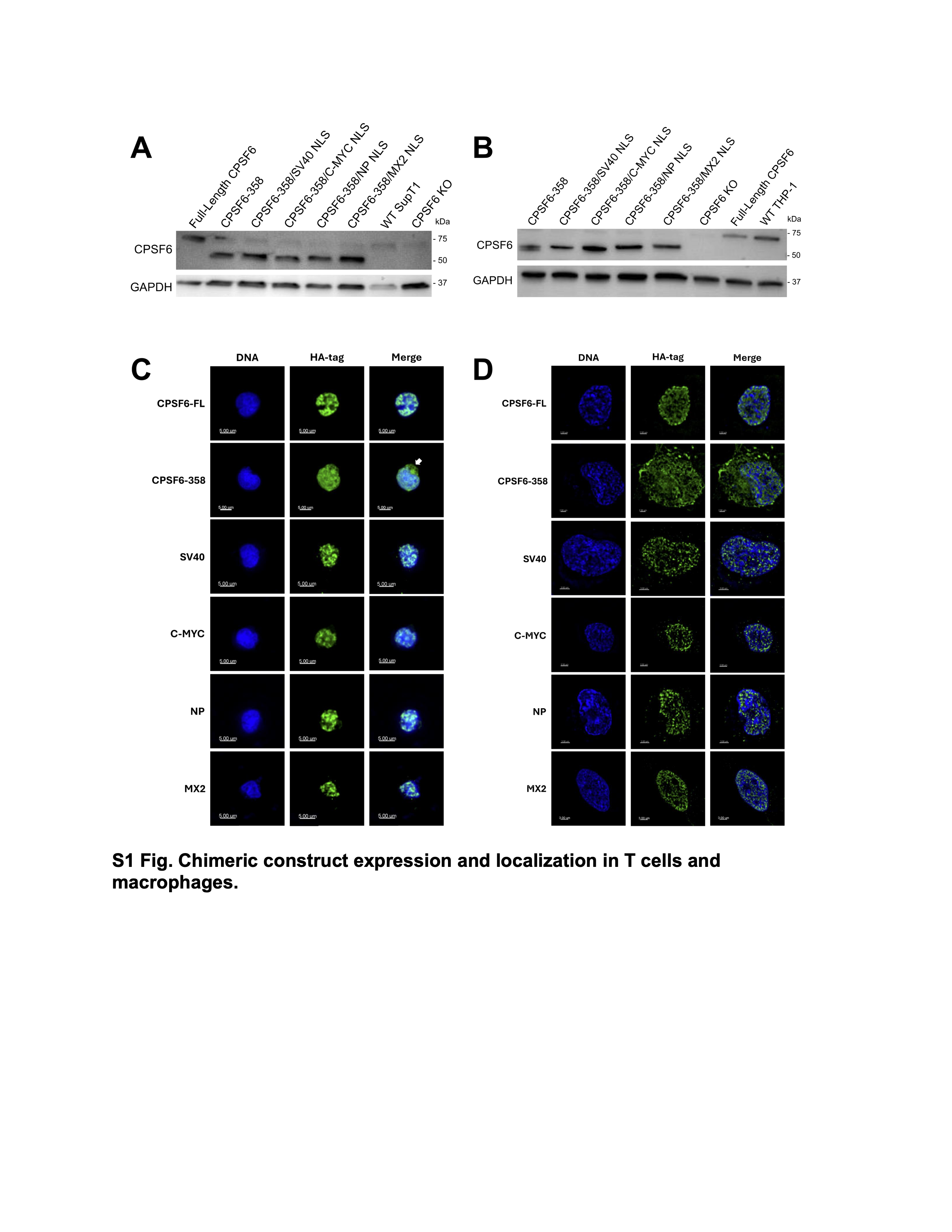

Supplement: S1 Fig — Western blot analysis of CPSF6-depleted stably-transduced SupT1 (A) and THP-1 (B) cell lines using anti-CPSF6 antibody to detect CPSF6-NLS construct expression following 48 h of doxycycline induction. Anti-GAPDH antibody used as loading control. Immunofluorescent imaging of SupT1 cell lines (C) and THP-1 cell lines differentiated into macrophages (D) using anti-HA antibody. Partial cytoplasmic staining of CPSF6-358 is evident in both cell types (highlighted in SupT1 cells by white arrow). (TIFF) [file ppat.1012354.s001.tiff]

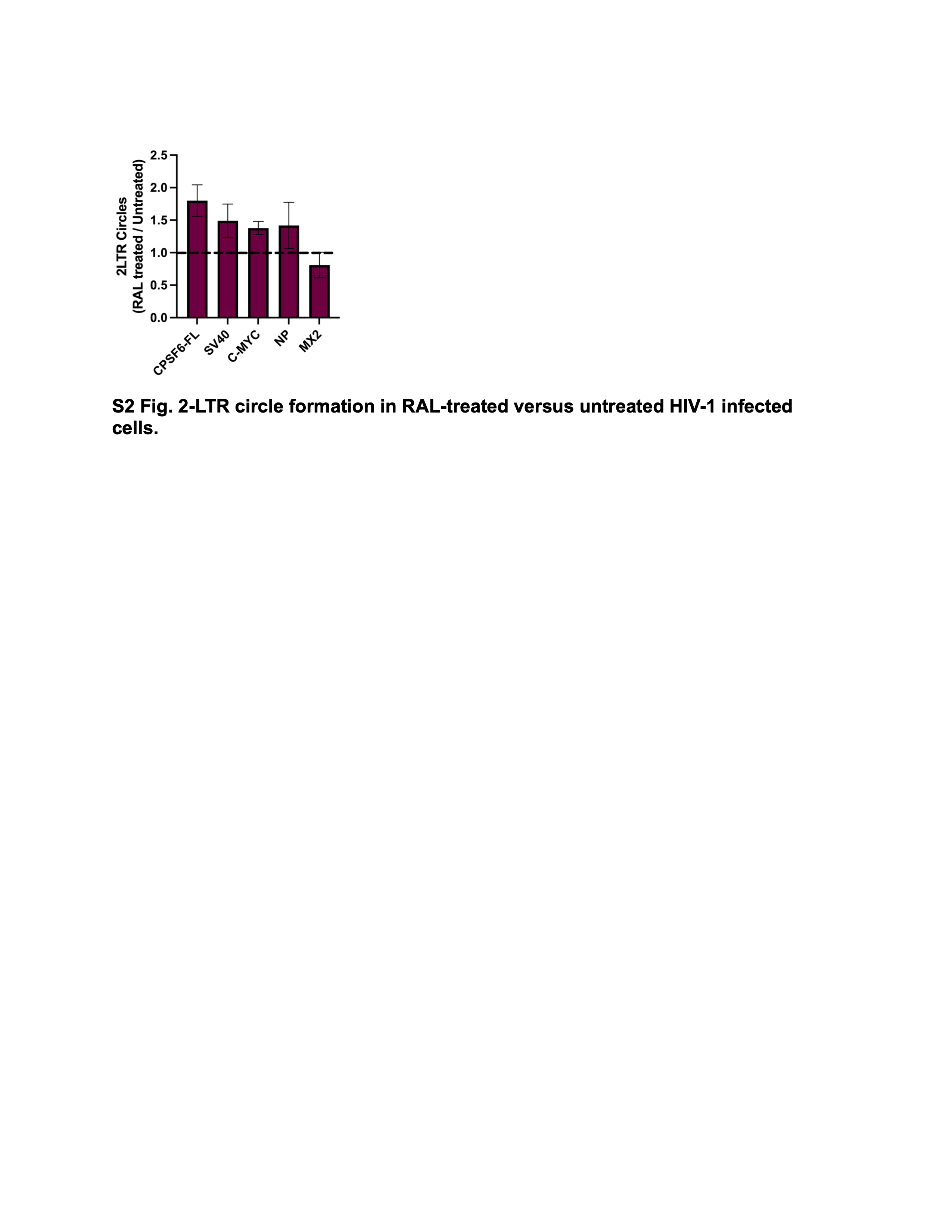

Supplement: S2 Fig — qPCR analysis was performed on genomic DNA isolated from WT HIV-1 infected cells in the presence or absence of raltegravir (RAL). No significant differences in 2-LTR circles were noted among tested cell lines relative to untreated HIV-1 infected cells. Results (mean ± SEM) are representative from 3 independent experiments with at least technical duplicates. (TIFF) [file ppat.1012354.s002.tiff]

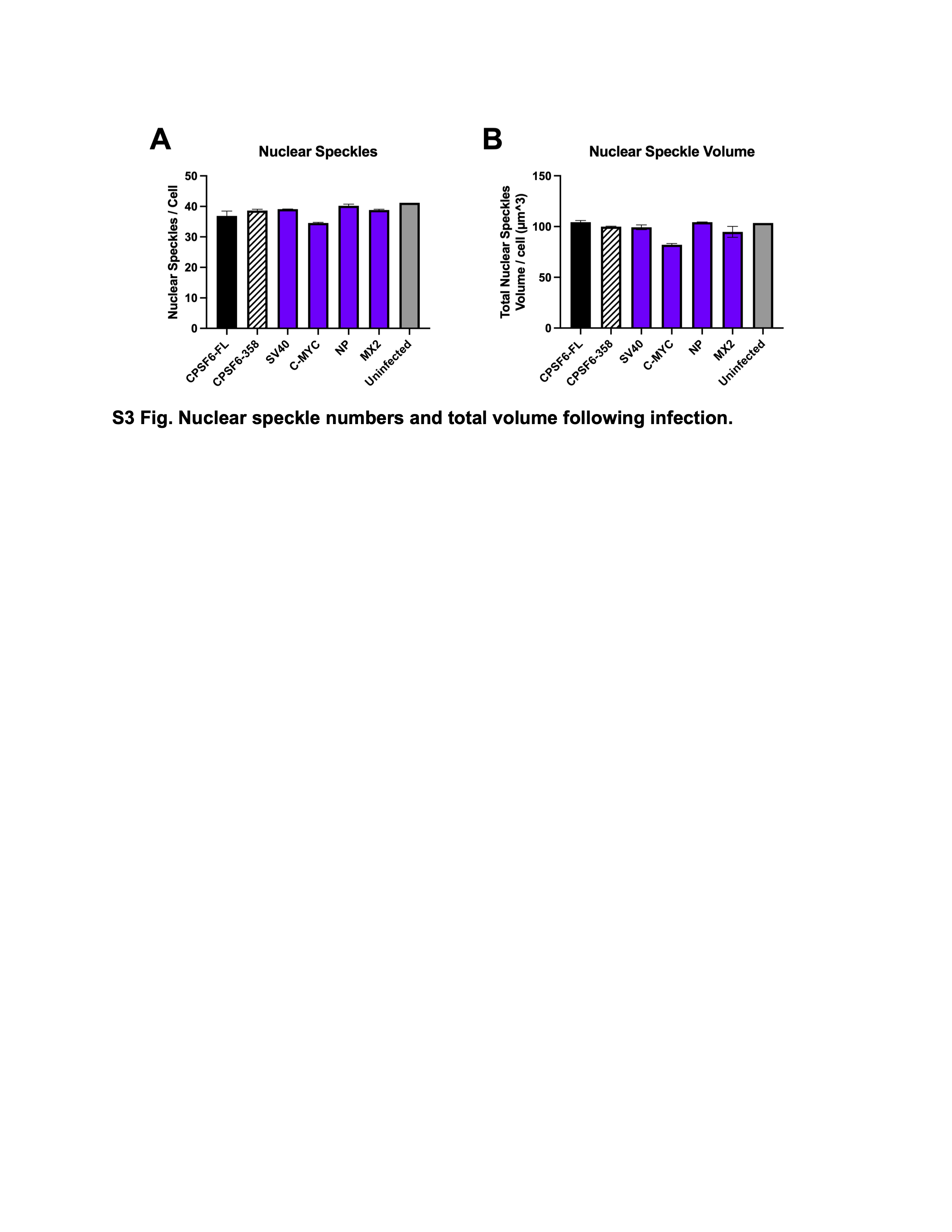

Supplement: S3 Fig — WT HIV-1 infected HeLa cells were fixed 24 h post-infection and stained for the nuclear speckle marker SC35. (A) IMARIS software was used to form surfaces around SC35 staining in the nucleus. Total SC35 surfaces per cell was determined and no significant differences were observed among the tested cell lines relative to uninfected control cells. (B) IMARIS software was utilized to determine the total volume of SC35 surfaces per cell. No significant differences among the tested HIV-1 infected cell lines relative to uninfected control cells were observed. At least 10 images were taken for each condition across 3 independent experiments. (TIFF) [file ppat.1012354.s003.tiff]

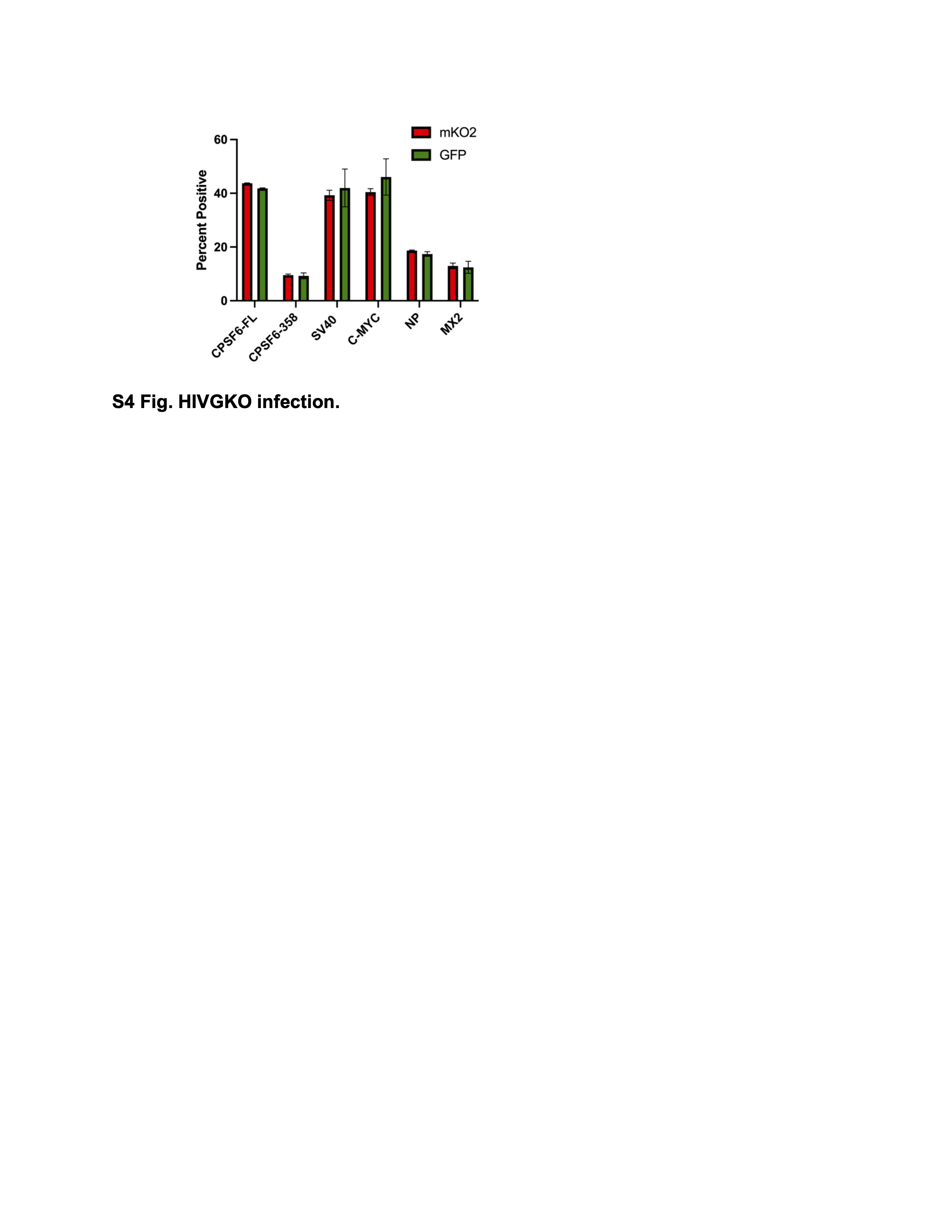

Supplement: S4 Fig — HeLa cells infected with dual reporter HIVGKO virus. mKO2 reporter is constitutive and GFP reporter is under control of the HIV LTR promoter. Comparable levels of mKO2 and GFP in the tested cell lines indicates that relative integration levels determined viral gene expression. (TIFF) [file ppat.1012354.s004.tiff]
