# Supplementary material for: The nuclear localization signal of CPSF6 governs post-nuclear import steps of HIV-1 infection
Source: PLoS Pathog. 2025 Jan 17;21(1):e1012354. doi: 10.1371/journal.ppat.1012354 (PMC11844840; doi:10.1371/journal.ppat.1012354)
Supplement: S1 Table — aResults (weighted mean ± weighted SD) are from ≥ two independent infection experiments. bRIC–random integration control. (DOCX) [file ppat.1012354.s005.docx]

**S1 Table. Integration distributions in CPSF6-NLS chimera HeLa cells^a^**

| **Sample^b^** | **N** | **Total**  **Sites** | **% Integration in Genes** | **% LAD Integration** | **% SPAD Integration** | **Gene Density Surrounding Integration Site** | **% Alphoid Integration** | **% LINE1 Integration** |
| --- | --- | --- | --- | --- | --- | --- | --- | --- |
| *CPSF6-FL* | 2 | 6958 | 70.68 ± 1.16 | 35.58 ± 0.04 | 18.07 ± 1.28 | 15.35 ± 0.56 | 0.1 ± 0.04 | 17.35 ± 0.14 |
| *SV40* | 4 | 4077 | 70.17 ± 0.8 | 35.61 ± 1.46 | 16.38 ± 1.23 | 14.67 ± 0.4 | 0.1 ± 0.04 | 18.94 ± 1.77 |
| *C-MYC* | 2 | 10530 | 68.33 ± 1.44 | 41.16 ± 1.42 | 12.66 ± 0.26 | 12.95 ± 0.31 | 0.11 ± 0.06 | 17.98 ± 0.9 |
| *NP* | 2 | 1159 | 62.99 ± 3.4 | 41.16 ± 1.74 | 11.04 ± 1.31 | 11.6 ± 0.72 | 0.43 ± 0.01 | 20.62 ± 3.03 |
| *MX2* | 2 | 925 | 63.78 ± 1.42 | 43.68 ± 0.11 | 9.41 ± 3.27 | 11.03 ± 1.55 | 0 ± 0 | 20.76 ± 1.83 |
| *RIC* | 5 | 926091 | 45.63 ± 0.01 | 47.98 ± 0.09 | 5.23 ± 0.05 | 9.18 ± 0.01 | 0.12 ± 0 | 16.97 ± 0.08 |
