# Supplementary material for: The nuclear localization signal of CPSF6 governs post-nuclear import steps of HIV-1 infection
Source: PLoS Pathog. 2025 Jan 17;21(1):e1012354. doi: 10.1371/journal.ppat.1012354 (PMC11844840; doi:10.1371/journal.ppat.1012354)
Supplement: S2 Table — aC6FL–CPSF6-FL. RIC–random integration control. (DOCX) [file ppat.1012354.s006.docx]

**S2 Table. Integration site statistics in CPSF6-NLS chimera HeLa cells**

|  | **Genes** | | **SPADs** | | **LADs** | | **Alphoid** | | **LINE1** | | **Gene Density** |
| --- | --- | --- | --- | --- | --- | --- | --- | --- | --- | --- | --- |
| **Comparison^a^** | **p-value** | **Odds Ratio** | **p-value** | **Odds Ratio** | **p-value** | **Odds Ratio** | **p-value** | **Odds Ratio** | **p-value** | **Odds Ratio** | **p-value** |
| *C6FL-SV40* | 0.6171 | 1.0246 | 0.0326 | 1.1252 | 1.0000 | 0.9987 | 1.0000 | 1.0254 | 0.0696 | 0.8985 | 0.3016 |
| *C6FL-C-MYC* | 0.0015 | 0.8949 | 0.0000 | 0.6574 | 0.0000 | 1.2662 | 1.0000 | 1.1329 | 0.3437 | 1.0443 | 0.0566 |
| *C6FL-NP* | 0.0000 | 1.4167 | 0.0000 | 1.7760 | 0.0005 | 0.7899 | 0.0936 | 0.2324 | 0.0277 | 0.8079 | 0.0321 |
| *C6FL-MX2* | 0.0000 | 1.3688 | 0.0000 | 2.1238 | 0.0000 | 0.7124 | 1.0000 | 0.0000 | 0.0368 | 0.8012 | 0.1268 |
| *C6FL-RIC* | 0.0000 | 2.8718 | 0.0000 | 3.9914 | 0.0000 | 0.5990 | 1.0000 | 0.8690 | 0.4614 | 1.0266 | 0.0410 |
| *SV40-RIC* | 0.0000 | 2.8027 | 0.0000 | 3.5472 | 0.0000 | 0.5997 | 1.0000 | 1.1800 | 0.2912 | 1.1122 | 0.0001 |
| *C-MYC-RIC* | 0.0000 | 2.5699 | 0.0000 | 2.6238 | 0.0000 | 0.7584 | 1.0000 | 0.9845 | 0.0277 | 1.0721 | 0.0368 |
| *NP-RIC* | 0.0000 | 2.0270 | 0.0000 | 2.2475 | 0.0000 | 0.7583 | 0.0682 | 3.7387 | 0.0076 | 0.8752 | 0.1316 |
| *MX2-RIC* | 0.0000 | 2.0980 | 0.0000 | 1.8794 | 0.0146 | 0.8408 | 1.0000 | 0.0000 | 0.0133 | 1.2813 | 0.3415 |
