# Supplementary material for: The nuclear localization signal of CPSF6 governs post-nuclear import steps of HIV-1 infection
Source: PLoS Pathog. 2025 Jan 17;21(1):e1012354. doi: 10.1371/journal.ppat.1012354 (PMC11844840; doi:10.1371/journal.ppat.1012354)
Supplement: S3 Table — aComparisons highlighted in red are underpowered. bC6FL–CPSF6-FL. RIC–random integration control. cRequired sample sizes to achieve Power = 0.8 with Alpha = 0.05 for left (n1) and right (n2) side of comparison as stated in comparison column. (DOCX) [file ppat.1012354.s007.docx]

**S3 Table. Power analysis of integration site statistics^a^**

|  |  | |  | **Genes** | | **SPADs** | | **LADs** | | **Alphoid** | | **LINE1** | |
| --- | --- | --- | --- | --- | --- | --- | --- | --- | --- | --- | --- | --- | --- |
| **Comparison^b^** | **Allocation Ratio** | **Power** | **Alpha (two-sided)** | **n1^c^** | **n2** | **n1** | **n2** | **n1** | **n2** | **n1** | **n2** | **n1** | **n2** |
| *C6FL-NP* | 6.0035 | 0.8 | 0.05 | 2014 | 336 | 1451 | 242 | 4146 | 691 | 10382 | 1730 | 7725 | 1287 |
| *C6FL-MX2* | 7.5221 | 0.8 | 0.05 | 3034 | 404 | 1121 | 150 | 2407 | 320 | 38387 | 5104 | 8670 | 1153 |
| *NP-RIC* | 0.0013 | 0.8 | 0.05 | 64 | 50820 | 146 | 116350 | 418 | 333677 | 1494 | 1193039 | 872 | 696512 |
| *MX2-RIC* | 0.0010 | 0.8 | 0.05 | 58 | 58023 | 268 | 268260 | 1055 | 1055638 | 3406 | 3409360 | 812 | 812331 |
